# *Lachnospira eligens* attenuates epileptogenesis via gut-brain axis regulation of blood-brain barrier integrity and neuroinflammation

**DOI:** 10.7150/thno.116959

**Published:** 2026-01-01

**Authors:** Huifeng Li, Ruili Niu, Wenzhen He, Huanling Lai, Shangnan Zou, Qihang Zou, Yue Gui, Tengyue Zhang, Guoyun Feng, Yue Xing, Dehai Gou, Xiaofeng Yang

**Affiliations:** 1 Department of Neurology, The First Affiliated Hospital, Guangzhou Medical University, Guangzhou 510120, China.; 2 Department of Basic Research, Guangzhou National Laboratory, Guangzhou 510005, China.; 3 Department of Neurology, The First Affiliated Hospital of Shantou University Medical College, Shantou 515041, China.; 4 Department of Neurology, Sun Yat-sen Memorial Hospital, Sun Yat-sen University, Guangzhou 510120, China.; 5 Department of Neurology, The Seventh Affiliated Hospital, Sun Yat-sen University, Shenzhen 518107, China.

**Keywords:** gut-brain axis, epilepsy, *Lachnospira eligens*, blood-brain barrier, neuroinflammation

## Abstract

**Rationale:** Emerging evidence implicates the gut microbiota in epilepsy pathogenesis through the microbiota-gut-brain axis, yet the functional contribution of specific microbial taxa to epileptogenesis remains unclear. This study aimed to investigate whether *Lachnospira eligens* (*L. eligens*) can alleviate epileptic activity by modulating the gut-brain axis, with a focus on intestinal barrier integrity, blood-brain barrier (BBB) integrity, and neuroimmune responses.

**Methods:** Using a cobalt wire-induced rat epilepsy model, we performed fecal 16S rDNA sequencing to assess gut microbiota alterations. Rats received daily oral gavage of *L. eligens* or PBS for 15 days, with colonization confirmed by qPCR. Seizure activity was monitored using long-term video electroencephalogram (EEG) and Racine scores. Barrier function, systemic inflammation, and microglial activation were assessed using FITC-dextran (FD**-**4, 4 kDa) assay, Western blotting (WB), immunohistochemistry (IHC), immunofluorescence (IF), ELISA, and qPCR. Serum short-chain fatty acids (SCFAs) were measured by liquid chromatography-tandem mass spectrometry (LC-MS/MS).

**Results:** Epileptic rats exhibited early gut microbiota dysbiosis, with a significant decline in *Lachnospira* abundance both preceding and succeeding seizure onset (*P* = 0.041, *P* = 0.026). *L. eligens* stably colonized the gut (Day 6 and Day 15, both *P <* 0.001). Supplementation significantly reduced grade 4-5 seizure frequency (*P* = 0.002) and prolonged seizure latency (*P* = 0.005). Barrier integrity improved, as indicated by lower plasma FD**-**4 (*P* < 0.001), increased colonic (WB: *P* = 0.013; IHC: *P* = 0.003) and cortical occludin expression (WB: *P* = 0.002; IHC: *P* = 0.01), and decreased serum lipopolysaccharide-binding protein (LBP) (*P* = 0.011). Neuroinflammation was attenuated, including reduced microglial activation (*P* = 0.048), lower pro-inflammatory cytokines (IL-1β, *P* = 0.047; IL-6, *P =* 0.001; TNF-α, *P =* 0.002), and decreased M1 polarization (*P* = 0.004). Serum butyrate increased (*P* = 0.014), and SCFAs, especially butyrate, suppressed lipopolysaccharide (LPS)-induced iNOS (*P* = 0.031) in BV2 cells.

**Conclusions:** These findings demonstrate that* L. eligens* mitigates epileptic activity by restoring intestinal barrier and BBB integrity and suppressing neuroinflammation. Our study highlights *L. eligens* as a promising microbiota-based intervention for epilepsy through modulation of the gut-brain axis.

## Introduction

Epilepsy is a chronic neurological disorder characterized by recurrent unprovoked seizures resulting from abnormal neuronal excitability and hypersynchronous network activity 1. While genetic, structural, infectious, metabolic, and immune-related etiologies have been extensively studied, the precise mechanisms underlying epileptogenesis remain incompletely understood 2, 3. Emerging evidence highlights the critical role of systemic and environmental factors—particularly the gut microbiota, which plays an important role in modulating central nervous system (CNS) function and influencing susceptibility to epilepsy through the gut-brain axis, a bidirectional communication system integrating neural, immune, endocrine, and metabolic signaling 3-11.

Alterations in the gut microbiota composition have been increasingly associated with neurological and psychiatric disorders, including epilepsy 12-14. Preclinical and clinical studies have reported gut microbial dysbiosis in individuals with epilepsy, often accompanied by reduced microbial diversity, increased pro-inflammatory taxa, and compromised intestinal barrier function 15-19. Such dysregulation can promote systemic inflammation and impair BBB integrity, thereby facilitating peripheral immune cell infiltration and microglial activation within the CNS 20, 21.

Recently, one proposed mechanistic pathway involves microbial translocation from the gut lumen into the systemic circulation, driven by increased intestinal permeability. Circulating endotoxins such as LPS can activate Toll-like receptor (TLR) 4 signaling, promoting microglial polarization toward a pro-inflammatory M1 phenotype 22. This phenotype is characterized by the release of cytokines, including IL-1β, IL-6 and TNF-α, which exacerbate neuroinflammation, disrupt synaptic homeostasis, and lower the threshold for seizure generation 23-25. Indeed, neuroinflammation mediated by activated microglia has been increasingly recognized as a key contributor to both the onset and progression of epilepsy 26-28.

Despite these insights, the functional contribution of specific gut microbial taxa to epileptogenesis remains poorly defined. Among candidate commensals, *Lachnospira*, a genus of SCFA-producing bacteria, has emerged as a potential neuroprotective taxon. *Lachnospira* species, including *L. eligens*, are known to enhance intestinal epithelial integrity, modulate immune responses, and maintain homeostasis through the production of butyrate and acetate. Notably, *Lachnospira* abundance has been reported to decrease in patients with epilepsy, yet the therapeutic potential of targeted restoration of this genus remains unexplored 29, 30. We hypothesize that the imbalance of intestinal flora disrupts intestinal homeostasis, thereby impairing intestinal barrier integrity and allowing translocation of pro-inflammatory microbial components such as LPS into systemic circulation 31, 32. Elevated LPS levels trigger systemic inflammation, which can disrupt the BBB and permit peripheral immune signals to enter the CNS 33-35. This promotes activation of microglia, the brain's resident immune cells, favoring a pro-inflammatory M1 phenotype that exacerbates neuronal hyperexcitability and epileptogenesis 23.

In this study, we employed a cobalt wire-induced rat epilepsy model, which replicates the dynamic phases of seizure development. Using a multi-modal approach including 16S rDNA sequencing, video-EEG monitoring, western blotting, immunohistochemistry, ELISA, immunofluorescence, and quantitative PCR, we investigated the temporal evolution of microbial dysbiosis and evaluated the impact of *L. eligens* on intestinal integrity, systemic inflammation, neuroimmune activation, and seizure severity. Our findings revealed a significant negative correlation between *Lachnospira* abundance and epileptic activity. Supplementation with *L. eligens* attenuated seizure severity, restored gut microbiota homeostasis, enhanced BBB integrity, and suppressed neuroinflammation by inhibiting microglial activation. To our knowledge, this is the first study to demonstrate that oral gavage with *L. eligens* can alleviate epileptogenesis through concurrent modulation of gut, immune, and neural barriers. These results provide mechanistic insight into the role of gut microbial modulation in epilepsy and identify *L. eligens* as a promising microbiota-based therapeutic candidate targeting the gut-immune-brain axis.

## Materials and Methods

### Animals and experimental design

Adult male Sprague-Dawley rats weighing 250-350 g were obtained and housed in a specific pathogen-free (SPF) barrier facility under standard laboratory conditions, including a 12 h light/dark cycle, ad libitum access to sterilized food and water, controlled temperature and humidity, and standardized cage-change and handling schedules across groups. No antibiotics, prebiotics, or additional probiotics were administered outside the planned *L. eligens* intervention. Animals were randomly assigned to experimental groups, and inclusion/exclusion criteria were based on health status, normal baseline behavior, and absence of overt disease. All experimental procedures were approved by the Ethics Committee of Guangzhou National Laboratory (No. 2024-07-A06).

To investigate gut microbiota alterations during epileptogenesis and epilepsy development, a pilot cohort was enrolled and rats were assigned to sham (n = 6) and cobalt-induced epilepsy (n = 6) groups.

In a second cohort, the sample size for evaluating the therapeutic effect of *L. eligens* (LE) was determined a priori using G*Power 3.1.9.4. Based on an anticipated effect size of 1.2, a significance level (α) of 0.05, and a desired power (1-β) of 0.8, 12 animals per group were required for the assessment of latency and generalized seizure outcomes (two-tailed test). Rats were then randomly assigned to three groups: Sham + PBS (n = 12), Cobalt + PBS (n = 12), and Cobalt + LE (n = 12). LE (1 × 10⁹ CFU/day) or vehicle (PBS) was administered via oral gavage for 15 consecutive days (Figure 3A).

### Surgical procedure and electrode implantation

**Figure 1:**
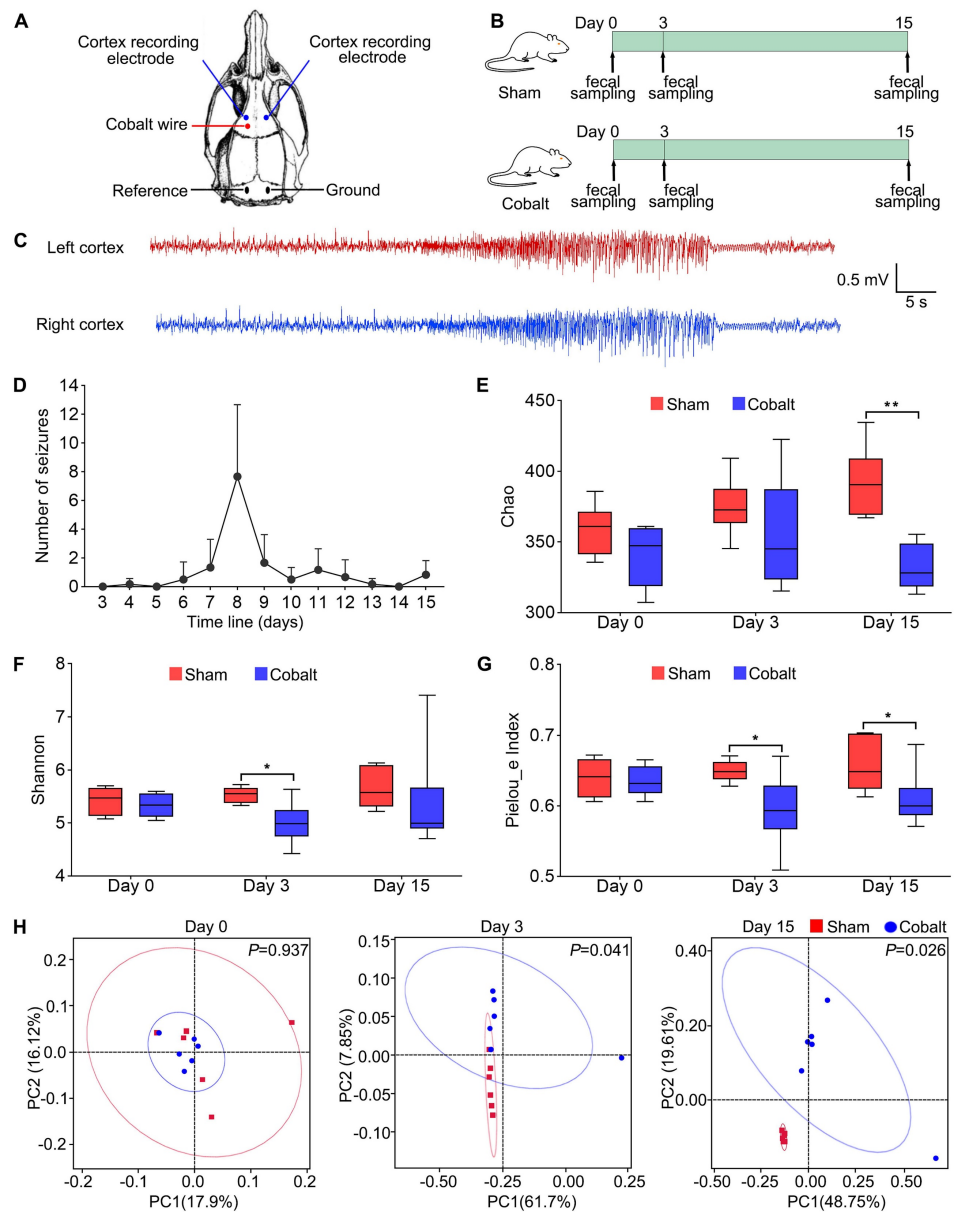
** Experimental design and gut microbial diversity changes during epileptogenesis and epilepsy development.** (A) Stereotaxic coordinates and representative locations of EEG electrodes and cobalt wire implantation in the motor cortex. (B) Schematic of the experimental design. Rats were randomly assigned to two groups: sham group (Sham, n = 6) and epilepsy group (Cobalt, n = 6). Fecal samples were collected at three time points: baseline (prior to cobalt wire implantation), day 3 post-implantation (epileptogenesis stage), and day 15 post-implantation (epileptic stage). (C) Representative electrographic recording demonstrating seizures. (D) Number of spontaneous seizures over time following cobalt wire insertion. (E-G) Alpha diversity was assessed using the Chao, Shannon and Pielou's evenness indices. (H) Beta diversity was evaluated using principal coordinates analysis (PCoA) based on unweighted UniFrac distances, showing distinct clustering of microbial communities between the sham and epilepsy groups. Mann-Whitney U test (E-G) and permutational MANOVA (H) were used. *p < 0.05, **p < 0.01, vs. the sham group.

Rats were anesthetized with 5% isoflurane for induction and maintained on 2% during surgery. Animals were positioned in a stereotaxic frame (RWD Life Science, Shenzhen, China). A sterile stainless-steel wire (1.0 mm diameter, 1.5 mm length) was implanted into the left motor cortex (AP +2.0 mm, ML +2.5 mm) in sham rats, while a sterile cobalt wire (1.0 mm diameter, 1.5 mm length; Sigma-Aldrich, St. Louis, MO, USA) was implanted into epilepsy rats at the same coordinates. Two screw electrodes were implanted bilaterally on the motor cortex (AP +3.0 mm, ML ±2.5 mm), with reference and ground electrodes placed above the cerebellum (Figure 1A).

At the end of the experiment, rats were deeply anesthetized. Cardiac blood was collected via puncture of the left ventricle using a sterile syringe. Euthanasia was then performed by decapitation to allow immediate brain collection. The cerebral cortex was rapidly dissected on ice and divided into two portions: one was snap-frozen in liquid nitrogen for molecular analysis, and the other was fixed in 4% paraformaldehyde (PFA) for histological examination. Subsequently, the abdominal cavity was opened under sterile conditions, and the colon (from the cecum to the rectum) was carefully excised. Luminal contents were gently flushed with ice-cold sterile saline. The colonic tissue was then divided into two portions: one was snap-frozen in liquid nitrogen for molecular analysis, and the other was fixed in 4% PFA for histological evaluation.

### Long-term video-EEG recording and analysis

Post-surgery, rats were individually housed in video-EEG recording cages allowing free movement. Continuous video-EEG (sampling rate 2 kHz) was recorded using a PowerLab 8/35 system (Colorado Springs, Co, USA). Seizure activity was analyzed using LabChart 8 software. Behavioral seizure severity was assessed according to the Racine scale 36, which categorizes seizure manifestations into five stages: 1, staring and mouth clonus; 2, head nodding; 3, unilateral forelimb clonus; 4, rearing and bilateral forelimb clonus; and 5, rearing and falling. Based on this classification, stages 1-3 were defined as partial seizures, while stages 4-5 as generalized seizures. Two blinded observers independently reviewed the recordings.

### *L. eligens* cultivation, administration, and colonization assessment

*L. eligens* (DSM3376) was obtained from German Collection of Microorganisms and Cell Cultures (DSMZ) and cultured anaerobically in methanosarcina medium at 37 °C. Cultures were harvested by centrifugation (8,000 g, 4 °C, 10 min), washed with sterile anaerobic PBS, and resuspended in PBS at a final concentration of 1 × 10⁹ CFU per rat per day. The bacterial suspension was administered by oral gavage daily for 15 days, starting on the day of model induction.

To verify colonization efficiency, fecal samples were collected on days 0, 6, and 15, and bacterial DNA was extracted using the DNA Extraction Kit (Tiangen, #DP328) according to the manufacturer's instructions. Quantitative PCR targeting *L. eligens*-specific 16S rRNA sequences was performed to determine bacterial abundance and colonization stability. Primer sequences are listed in Table S1.

### Fecal collection and 16S rDNA sequencing

Fecal sampling was performed by a single trained handler in a quiet room, within a fixed time window each day. Handling was gentle and brief (less than 60 s per animal), and the sampling order was randomized. All groups and timepoints were handled identically. Fecal samples were collected in sterile EP tubes, from which total genomic DNA was extracted. The purity and concentration of the extracted DNA were assessed using 1% agarose gel electrophoresis. Subsequently, DNA samples were aliquoted into centrifuge tubes and diluted to a concentration of 1 ng/μL using sterile water. The V4 hypervariable region of the bacterial 16S rRNA gene was amplified using the primer pair 16S V4 515F-806R, where the forward primer 515F (GTGCCAGCMGCCGCGGTAA) and the reverse primer 806R (GGACTACHVGGGTWTCTAAT) were employed. PCR amplification was performed in a 30 μL reaction mixture containing 15 µL of Phusion® High-Fidelity PCR Master Mix (New England Biolabs), 0.2 µM of each primer, and 10 ng of genomic DNA template. The thermal cycling conditions consisted of an initial denaturation at 98 °C for 1 min, followed by 30 cycles of denaturation at 98 °C for 10 s, annealing at 50 °C for 30 s, and extension at 72 °C for 30 s. The PCR products were initially analyzed by 2% agarose gel electrophoresis. Following confirmation of successful amplification, target bands were excised and purified using the universal DNA purification and recovery kit (TianGen, #DP214). A sequencing library was prepared using the NEB Next® Ultra™ II FS DNA PCR-free Library Prep Kit (New England Biolabs, USA). The quality and quantity of the constructed library were assessed using Qubit and quantitative PCR. Finally, paired-end 250 bp sequencing was performed on the NovaSeq 6000 platform.

### FITC-dextran (FD-4) assay for intestinal permeability

Intestinal permeability was assessed by measuring the plasma flux of FD-4 (Sigma-Aldrich, USA). A freshly prepared FD-4 solution (50 mg/mL in PBS) was protected from light until use. Rats received FD-4 by oral gavage at a dose of 200 mg/kg body weight after a standard pretreatment protocol. Blood samples were collected and centrifuged to obtain plasma 4 h after administration. FD-4 fluorescence was then quantified at excitation/emission wavelengths of 490/525 nm using a microplate reader.

### Western blotting

Colonic, or cortical tissues, or cultured cells were lysed in RIPA lysis buffer (Beyotime, #P0013B) supplemented with a protease inhibitor cocktail (Selleck, #B14002). Equal protein amounts were separated by SDS-PAGE and transferred to polyvinylidene fluoride membranes (Merck Millipore, #IPVH00010). The membranes were blocked and incubated overnight at 4 °C with primary antibodies: anti-Occludin (1:10,000, Proteintech, #27260-1-AP), anti-TLR4 (1:1000, Cell Signaling Technology, #14358), anti-COX-2 (1:1000, Cell Signaling Technology, #12282), anti-Arginase-1 (1:1000, Cell Signaling Technology, #93668), anti-iNOS (1:1000, Cell Signaling Technology, #13120), and anti-GAPDH (1:10,000, Proteintech, #60004-1-AP), followed by species-specific horseradish peroxidase (HRP)-conjugated secondary antibodies, including rabbit HRP (1:10,000, Abcam, #ab6721) and murine HRP (1:10,000, Abcam, #ab205719) for 1 h at room temperature. Protein bands were visualized with a chemiluminescence imaging system.

### Immunohistochemistry

Paraffin-embedded brain and colon sections (6 μm for brain cortex; 4 μm for colon) were dewaxed, rehydrated, and subjected to antigen retrieval. Occludin expression was detected using an immunohistochemistry kit (keyGEN bioTECH, #KGOS300), with overnight incubation of primary antibody (1:10,000, Proteintech, #27260-1-AP) at 4 °C. The next day, slices were incubated with the secondary antibody at room temperature. Immunoreactivity was visualized using diaminobenzidine (DAB), and nuclei were counterstained with hematoxylin. The immunohistochemistry scoring system for occludin expression integrates both staining intensity and the percentage of positively stained cells for a more accurate evaluation. Staining intensity is classified into four levels: 0 points (negative, no staining), 1 point (weakly positive, light yellow), 2 points (positive, brownish yellow), and 3 points (strongly positive, dark brown). The percentage of positive cells is also categorized into four levels: 1 point (1%-25%), 2 points (26%-50%), 3 points (51%-75%), and 4 points (76%-100%). The final score was determined by multiplying the staining intensity score by the percentage score.

### Enzyme-linked immunosorbent assay (ELISA)

Serum levels of lipopolysaccharide-binding protein (LBP) were quantified using a commercial ELISA kit (CUSABIO, CSB-E11184r) according to the manufacturer's instructions. The kit was equilibrated at room temperature for 1 h prior to use. Serum samples were diluted 1:5 with the assay buffer, and 100 μL of the diluted sample was added to each well of a 96-well plate pre-coated with LBP-specific antibodies. After 2 h of incubation at 37 °C, the wells were washed and incubated with 100 μL of biotin-labeled detection antibody for 1 h, followed by HRP-conjugated avidin for another 1 h at 37 °C. After additional washes, the substrate solution was added and incubated at 37 °C in the dark for 15 min. The reaction was terminated with stop solution, and absorbance was immediately measured at 450 nm using a microplate reader.

### Immunofluorescence

Following dewaxing, rehydration, and antigenic retrieval, the brain sections were blocked with goat serum (1:20, Solarbio, #SL038). Primary antibodies were applied and incubated overnight at 4 °C: CD68 (1:1000, Cell Signaling Technology, #97778), Arginase-1 (1:100, Cell Signaling Technology, #93668), iNOS (1:100, Santa Cruz Biotechnology, sc-7271), Iba1 (Mouse monoclonal, 1:500, Abcam, #ab283319) and Iba1 (Rabbit monoclonal, 1:500, Cell Signaling Technology, #17198). After washing, sections were incubated with secondary antibody including goat anti-mouse (Alexa Fluor®555) (1:500, Abcam, ab150114) and goat anti-rabbit (Alexa Fluor®488) (1:500, Abcam, ab150077) at room temperature for 2 h. Finally, slices were mounted with DAPI-containing anti-fading mounting medium (Solarbio, S2110) and analyzed using fluorescence microscopy.

### RNA extraction and quantitative PCR

Total RNA was extracted from cortical tissues using the animal tissue RNA extraction kit (Tiangen, #DP431) according to the manufacturer's instructions. RNA concentration and purity were determined spectrophotometrically at 260/280 nm. cDNA was synthesized from 500 ng RNA using the FastKing RT Kit with gDNase (Tiangen, #KR118). Quantitative PCR was performed using the SuperReal PreMix Plus SYBR Green kit (Tiangen, #FP209) on a QuantStudio 3 system. Primer sequences are listed in Table S1.

Thermal cycling conditions were: 95 °C for 3 min, followed by 40 cycles of 95 °C for 5 s and 60 °C for 15 s.

### Targeted SCFA analysis

Serum SCFAs were quantified using LC-MS/MS. Briefly, serum samples were mixed with 80% methanol, vortexed, and centrifuged, and the resulting supernatant was derivatized at 40 °C for 40 min. The derivatized samples were then analyzed by LC-MS/MS for targeted quantification of SCFAs.

### BV2 cell culture and treatment

BV2 microglial cells were maintained in DMEM supplemented with 10% FBS and 1% penicillin-streptomycin at 37 °C in a humidified incubator with 5% CO2. Cells were pretreated with SCFAs (sodium acetate, 30 mM; sodium propionate, 1 mM; sodium butyrate, 1 mM) for 1 h, followed by stimulation with LPS (100 ng/mL) for 6 h. After treatment, cells were harvested for protein extraction and subsequent analyses.

### Statistical analysis

Statistical analyses were performed using SPSS 25.0 and GraphPad Prism 8.0.1. The normality of data distribution was assessed using the Shapiro-Wilk test. Normally distributed data were presented as mean ± standard deviation (SD) or as dot plots. Student's t*-*test was used for comparisons between two groups. For multiple group comparisons of normally distributed data, one-way analysis of variance (ANOVA) followed by Bonferroni or Dunnett's T3 post hoc test was performed. For non-normally distributed data, the Mann-Whitney U test was used for two-group comparisons, while the Kruskal-Wallis test followed by Dunn's post hoc test was used for multiple group comparisons. Family-wise error for multiple comparisons was controlled using the Bonferroni correction (two-sided α = 0.05). Statistical significance was set at *p* < 0.05.

## Results

### Epileptogenesis and epilepsy development are associated with gut microbiota dysbiosis and reduced *Lachnospira* abundance

To explore gut microbial dynamics during epileptogenesis and epilepsy progression, we established a cobalt wire-induced rat epilepsy model, which showed a 100% success rate with a latency to first seizure onset ranging from 4 to 8 days. Fecal samples were collected at baseline (pre-modeling), during epileptogenesis phase (day 3 post-surgery, prior to seizure onset), and post-epilepsy phase (day 15 post-surgery, after seizure onset) (Figure 1B). 16S rDNA sequencing revealed a significant decline in alpha diversity in the epilepsy group compared with the sham group. Specifically, the Chao index (Figure 1E) was significantly decreased at Day 15, the Shannon index (Figure 1F) showed a significant reduction at Day 3, and Pielou's evenness index (Figure 1G) was reduced during both the epileptogenesis and epilepsy stages. Principal coordinates analysis (PCoA) of beta diversity also showed significant microbial composition shifts (Figure 1H), indicating marked dysbiosis associated with epileptic progression.

**Figure 2:**
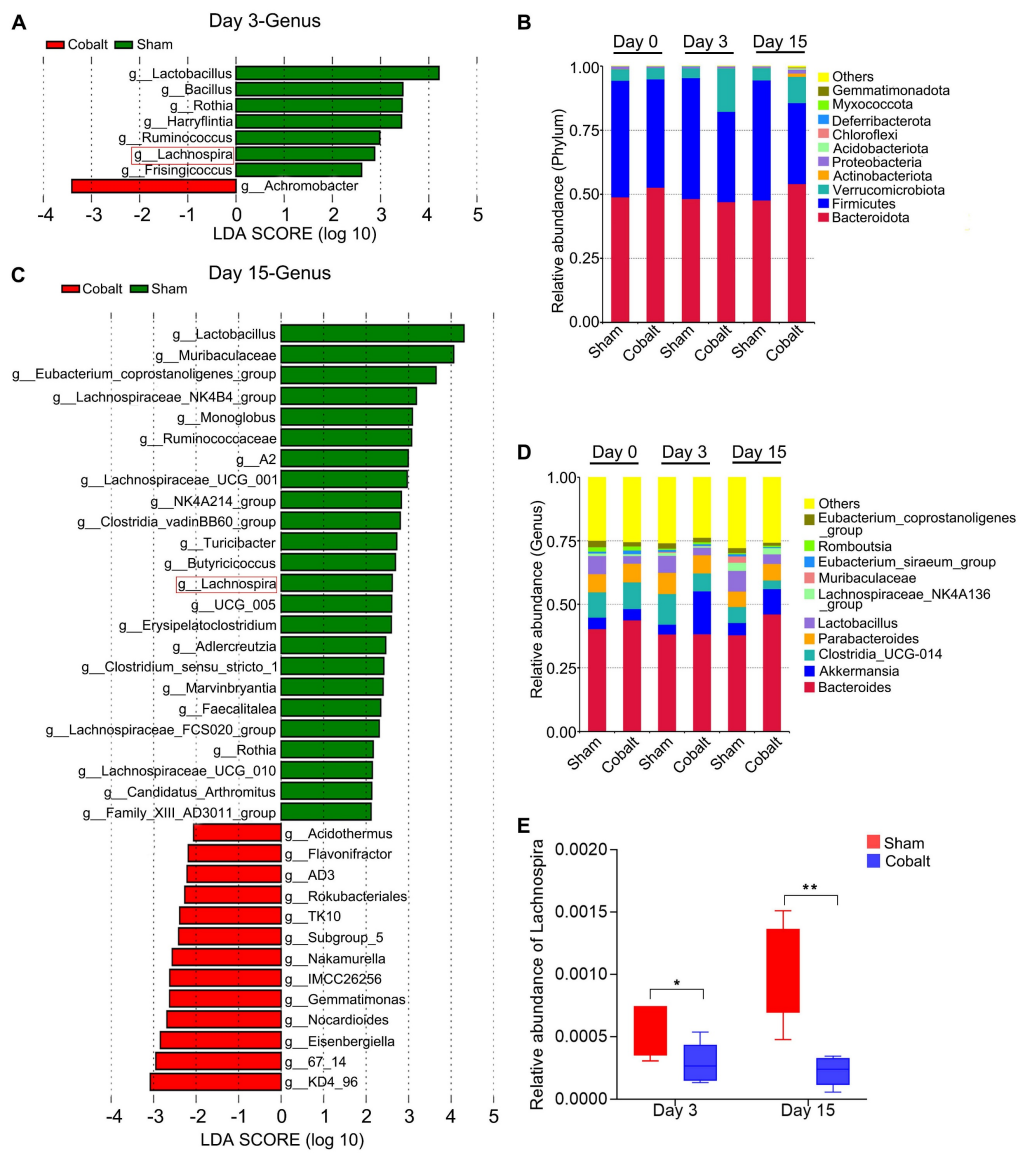
** Alterations in gut microbiota composition during epileptogenesis and epilepsy progression reveal a significant reduction in *Lachnospira* abundance.** (A) Discriminant taxa at the genus level between the sham and epilepsy groups on day 3 post-implantation. (B) Relative abundance of gut microbiota at the phylum level across groups. (C) Discriminant taxa at the genus level between the sham and epilepsy groups on day 15 post-implantation. (D) Relative abundance of gut microbiota at the genus level across groups. (E) *Lachnospira* abundance was significantly reduced in the epilepsy group during both the epileptogenesis (day 3) and epilepsy (day 15) stages compared to the sham group. Linear discriminant analysis (LDA) effect size (LEfSe) with a threshold of LDA score > 2 (A, C) and a two-tailed *t*-test (E) were used. *p < 0.05, **p < 0.01, vs. the sham group.

At the phylum level, relative abundances of *Verrucomicrobiota, Actinobacteriota, Proteobacteria*,* Acidobacteriota*, and *Chloroflexi* were elevated in the epilepsy group (Figure 2B). Genus-level analysis revealed increased *Akkermansia* and *Romboutsia,* while beneficial genera—including *Clostridia_UCG-014*, *Lactobacillus, Lachnospiraceae_NK4A136_group, Muribaculaceae, and Eubacterium* spp*.* were significantly reduced (Figure 2D). Linear discriminant analysis effect size (LEfSe) identified key taxa with altered abundance across timepoints (Figure 2A, C). Notably, *Lachnospira* abundance declined significantly in the epilepsy group during both the epileptogenesis and epilepsy stages (Figure 2E), suggesting a potential role in early epileptogenic susceptibility.

### *L. eligens* stably colonizes the gut and mitigates seizure severity

**Figure 3:**
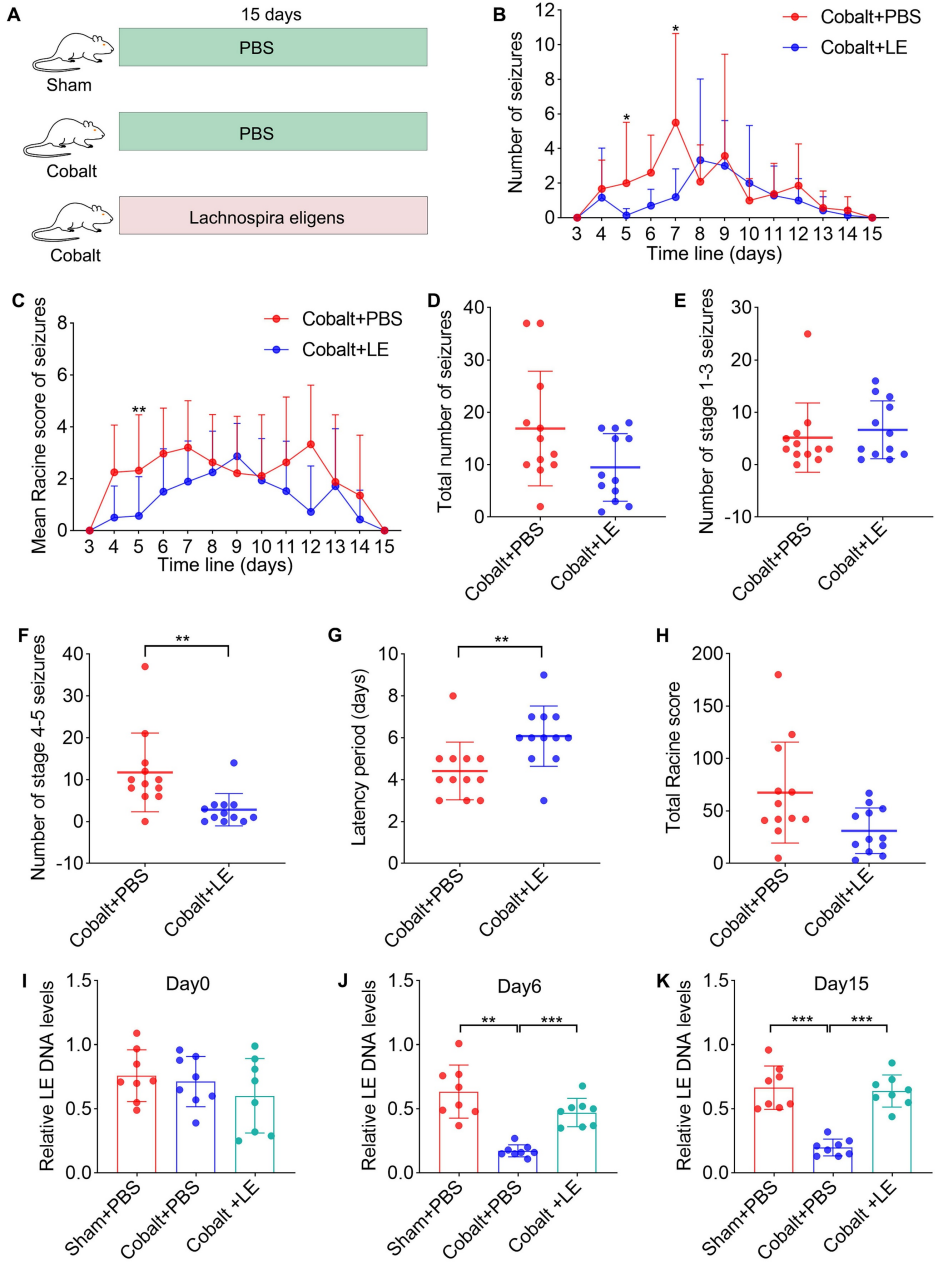
***L. eligens* stably colonizes the gut, attenuates seizure severity, and prolongs seizure latency in epileptic rats.** (A) Schematic of the experimental design. Rats were randomly assigned into three groups: sham-operated rats treated with PBS (Sham + PBS, n = 12), epilepsy model rats treated with PBS (Cobalt + PBS, n = 12), and epilepsy model rats treated with* L. eligens* (Cobalt + LE, n = 12). *L. eligens* (1 × 10⁹ CFU/day) or PBS was administered via oral gavage for 15 consecutive days. (B) Number of spontaneous seizures observed in the Cobalt + PBS and Cobalt + LE groups over time following cobalt wire insertion. (C) Mean Racine score in each group. (D) Total number of seizures. (E) Number of partial seizures (Racine stages 1-3) in each group. (F) Number of generalized seizures (Racine stages 4-5). (G) Seizure latency was significantly prolonged in the Cobalt + LE group compared to the Cobalt + PBS group. (H) Total Racine score. (I-K) Relative *L. eligens* DNA levels in each group on days 0, 6, and 15. Mann-Whitney U test (B-H) and One-way ANOVA followed by Bonferroni (I) and Dunnett's T3 post hoc test (J-K) were used. *p < 0.05, **p < 0.01, ***p < 0.001, vs. the Cobalt + PBS group.

To evaluate therapeutic efficacy, we administered *L. eligens* (1×10⁹ CFU/day) to epilepsy model rats via oral gavage for 15 days. Controls included the PBS-treated epilepsy and PBS-treated sham groups (Figure 3A). Fecal samples collected on Day 0, Day 6, and Day 15 were analyzed by quantitative PCR, confirming stable gut colonization with bacterial abundance significantly increased compared to the PBS-treated epilepsy group on Day 6 and Day 15 (Figure 3J-K).

Behavioral assessments using Racine scale revealed no significant differences in the total number of seizures (Figure 3D), the number of grade 1-3 partial seizures (Figure 3E), or the total Racine score (Figure 3H). However, the *L. eligens*-treated epilepsy group exhibited a marked reduction in grade 4-5 generalized seizures (Figure 3F) compared to the PBS-treated epilepsy group. Moreover, latency to first seizure was significantly prolonged in the *L. eligens*-treated epilepsy group (Figure 3G), indicating that *L. eligens* effectively delays seizure onset and reduces severity.

### *L. eligens* restores microbial homeostasis and enhances intestinal barrier integrity

**Figure 4:**
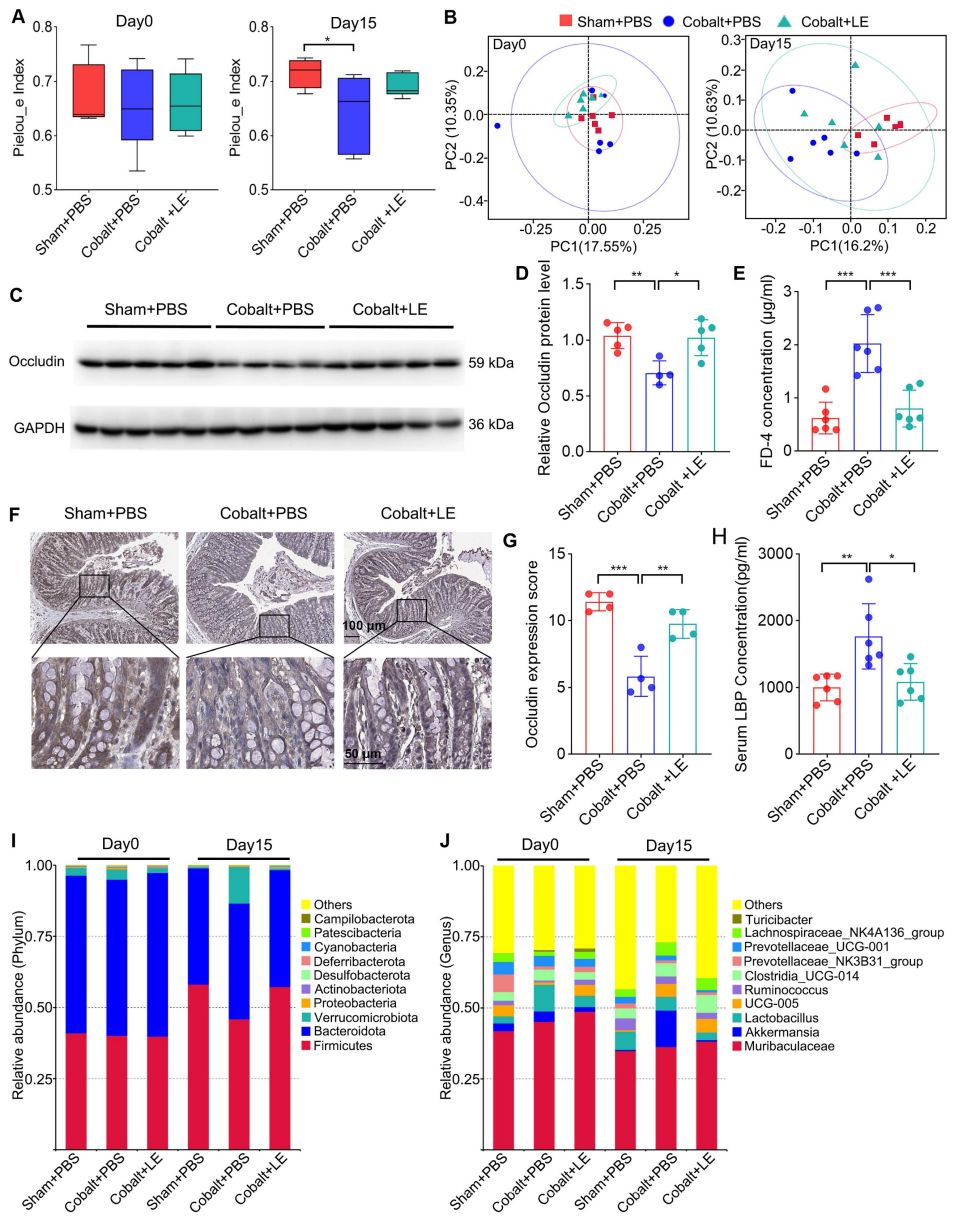
***L. eligens* supplementation restores gut microbiota homeostasis and enhances intestinal barrier integrity.** (A) Alpha diversity analysis among the Sham + PBS, Cobalt + PBS, and Cobalt + LE groups based on Pielou's evenness index. (B) Principal coordinates analysis (PCoA) showing beta diversity and clustering of microbial communities among the three groups. (C) Western blot analysis of occludin expression in colonic tissues. (D) Quantification of occludin band intensity using grayscale analysis with ImageJ software. (E) Plasma FD-4 concentration. (F) Representative immunohistochemical images of occludin expression in colonic sections. Occludin-positive staining appears as yellowish-brown deposits, while cellular nuclei are counterstained with hematoxylin (blue). Scale bar: 50 µm. (G) Histological scoring of occludin expression based on staining intensity and percentage of positive cells. (H) Serum LBP levels measured by ELISA in each group. (I) Relative abundance of gut microbiota at the phylum level across groups. (J) Relative abundance of gut microbiota at the genus level across groups. Data are presented as mean ± SD. One-way ANOVA followed by Bonferroni post hoc test (D, E, G, H) and Kruskal-Wallis test followed by Dunn's post hoc test (A) were used. *p < 0.05, **p < 0.01, ***p < 0.001, vs. the Cobalt + PBS group.

Post-intervention 16S rRNA sequencing showed that *L. eligens* treatment partially restored gut microbial diversity. Although alpha diversity in the treated epilepsy group did not reach statistical significance versus the PBS-treated epilepsy group, the trend approached that of the sham group (Figure 4A). Additionally, PCoA analysis revealed that the microbial composition in the *L. eligens*-treated epilepsy group resembled that of the PBS-treated sham group (Figure 4B).

Taxonomic analysis revealed decreased abundance of pro-inflammatory phyla such as *Verrucomicrobiota* and *Proteobacteria* following *L. eligens* treatment (Figure 4I). At the genus level, *Akkermansia*—previously elevated in the epilepsy group—was reduced after supplementation, approaching sham levels (Figure 4J).

To assess intestinal barrier integrity, plasma FD-4 levels were measured, revealing significantly elevated FD-4 in the PBS-treated epilepsy group compared with both the PBS-treated sham and *L. eligens*-treated epilepsy groups (Figure 4E). Consistently, Western blotting and immunohistochemistry revealed significantly higher occludin expression in the *L. eligens*-treated epilepsy group compared to PBS-treated epilepsy group (Figure 4C, D, F and G). Correspondingly, serum LBP levels, indicative of barrier disruption and systemic inflammation, were significantly lower in the L. eligens-treated epilepsy group (Figure 4H).

### *L. eligens* protects BBB integrity and suppresses microglial activation

**Figure 5:**
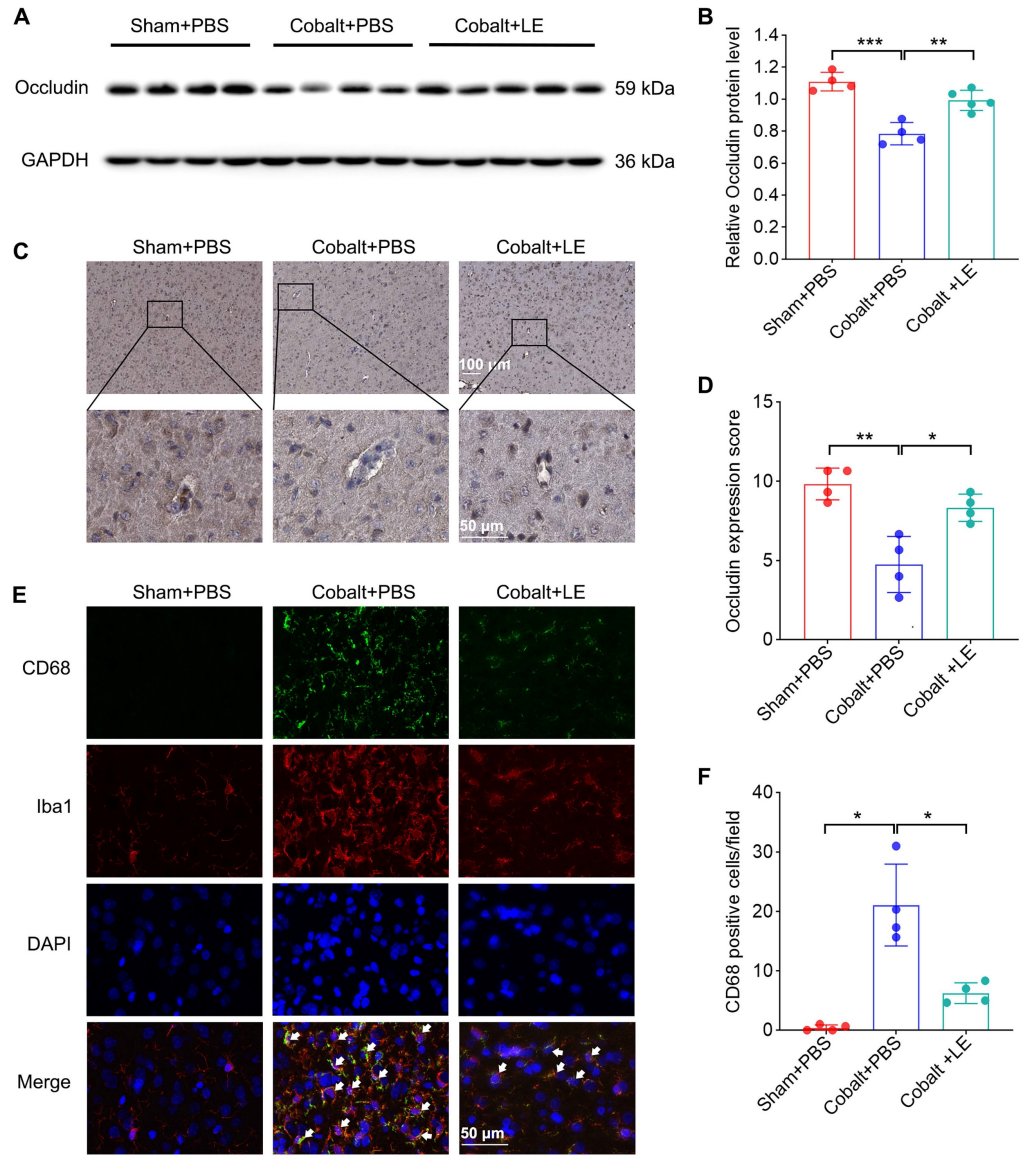
***L. eligens* supplementation preserves blood-brain barrier integrity and suppresses microglia activation.** (A) Western blot analysis of occludin expression in cortical tissues from Sham + PBS, Cobalt + PBS and Cobalt + LE groups. (B) Quantification of cortical occludin expression by grayscale densitometry using ImageJ software. (C) Representative immunohistochemical staining of occludin in the cortex. Occludin-positive staining appears as brownish-yellow deposits; nuclei were counterstained with hematoxylin (blue). (D) Histopathological scoring of cortical occludin protein expression based on staining intensity and percentage of positive cells. (E) Representative immunofluorescence images showing activated microglia in the cortex. CD68⁺ cells are shown in green, Iba1⁺ microglia in red, and DAPI-stained nuclei in blue. Yellow regions indicate co-localization of CD68 and Iba1 signals, demonstrating activated microglia. (F) Quantification of CD68⁺ microglia across experimental groups based on three representative immunofluorescence fields per sample. Scale bar: 50 µm. Data are presented as mean ± SD. One-way ANOVA followed by Bonferroni (B, D) and Dunnett's T3 post hoc test (F) were used. *p < 0.05, **p < 0.01, ***p < 0.001, vs. the Cobalt + PBS group.

Western blotting and immunohistochemical analyses revealed significantly reduced cortical occludin levels in the PBS-treated epilepsy group, which were restored in the *L. eligens*-treated group (Figure 5A-D). These findings suggest that *L. eligens* supplementation reinforces BBB integrity.

Immunofluorescence staining for CD68 and Iba1 revealed increased microglial activation in the PBS-treated epilepsy group, which was significantly attenuated following *L. eligens* administration (Figure 5E-F), indicating suppression of neuroinflammatory microglial responses.

### *L. eligens* inhibits M1 microglial polarization and reduces neuroinflammatory cytokine expression

**Figure 6:**
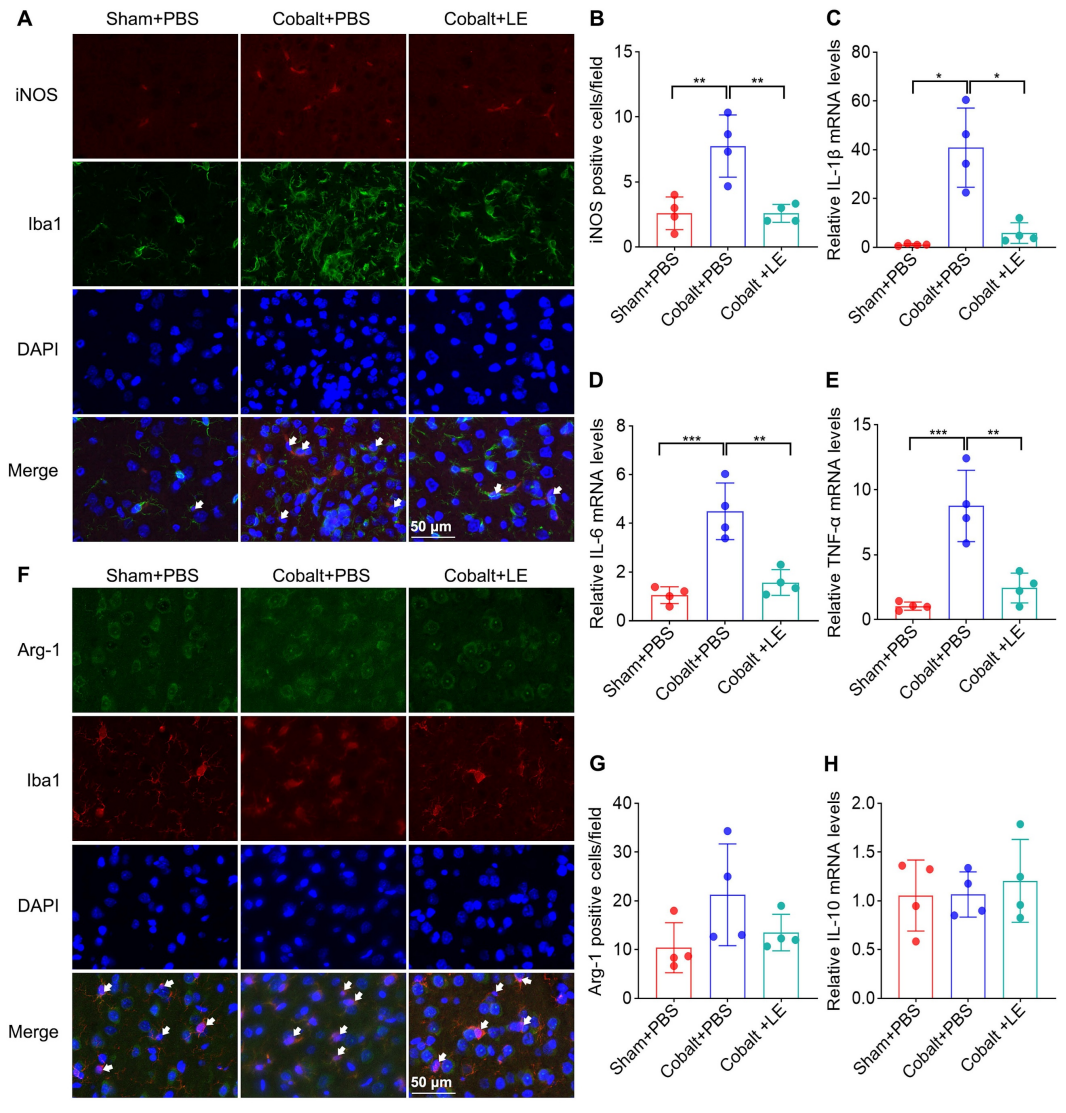
***L. eligens* treatment suppresses M1 microglial polarization and mitigates neuroinflammation.** (A) Representative immunofluorescence staining images showing M1 polarized microglia in the cortex. iNOS⁺ cells are shown in red, Iba1⁺ microglia in green, and DAPI-stained nuclei appear blue. Scale bar: 50 µm. (B) Quantification of M1 microglia (iNOS⁺/Iba1⁺) across experimental groups based on three representative immunofluorescence fields per sample. (C-E) Relative mRNA expression levels of IL-6, IL-1β, and TNF-α were calculated using the 2^-ΔΔCt method and normalized to GAPDH. (F) Representative immunofluorescence images showing M2-polarized microglia. Arg-1⁺ cells are shown in green, Iba1⁺ microglia in red, and DAPI-stained nuclei appear blue. (G) Quantification of M2 microglia (Arg-1⁺/Iba1⁺) in each group based on three representative immunofluorescence fields per sample. (H) Relative mRNA expression levels of IL-10 in each group. Scale bar: 50 µm. Data are presented as mean ± SD. One-way ANOVA followed by Bonferroni (B, D, E) and Dunnett's T3 post hoc test (C) were used. *p < 0.05, **p < 0.01, ***p < 0.001, vs. the Cobalt + PBS group.

To further explore microglial phenotype, we assessed M1/M2 polarization. Expression of M1 markers was significantly upregulated in the PBS-treated epilepsy group compared to the PBS-treated sham group, and was significantly reduced following *L. eligens* treatment (Figure 6A-B). Conversely, M2 marker expression and IL-10 levels showed no significant changes among groups (Figure 6F-H), suggesting a selective suppression of pro-inflammatory microglial responses.

Quantitative PCR revealed that IL-1β, IL-6, and TNF-α mRNA levels were markedly elevated in the PBS-treated epilepsy group but significantly suppressed in the *L. eligens*-treated epilepsy group (Figure 6C-E). These findings support the role of *L. eligens* in dampening pro-inflammatory signaling pathways implicated in seizure propagation.

### SCFAs suppress microglial pro-inflammatory activation

**Figure 7:**
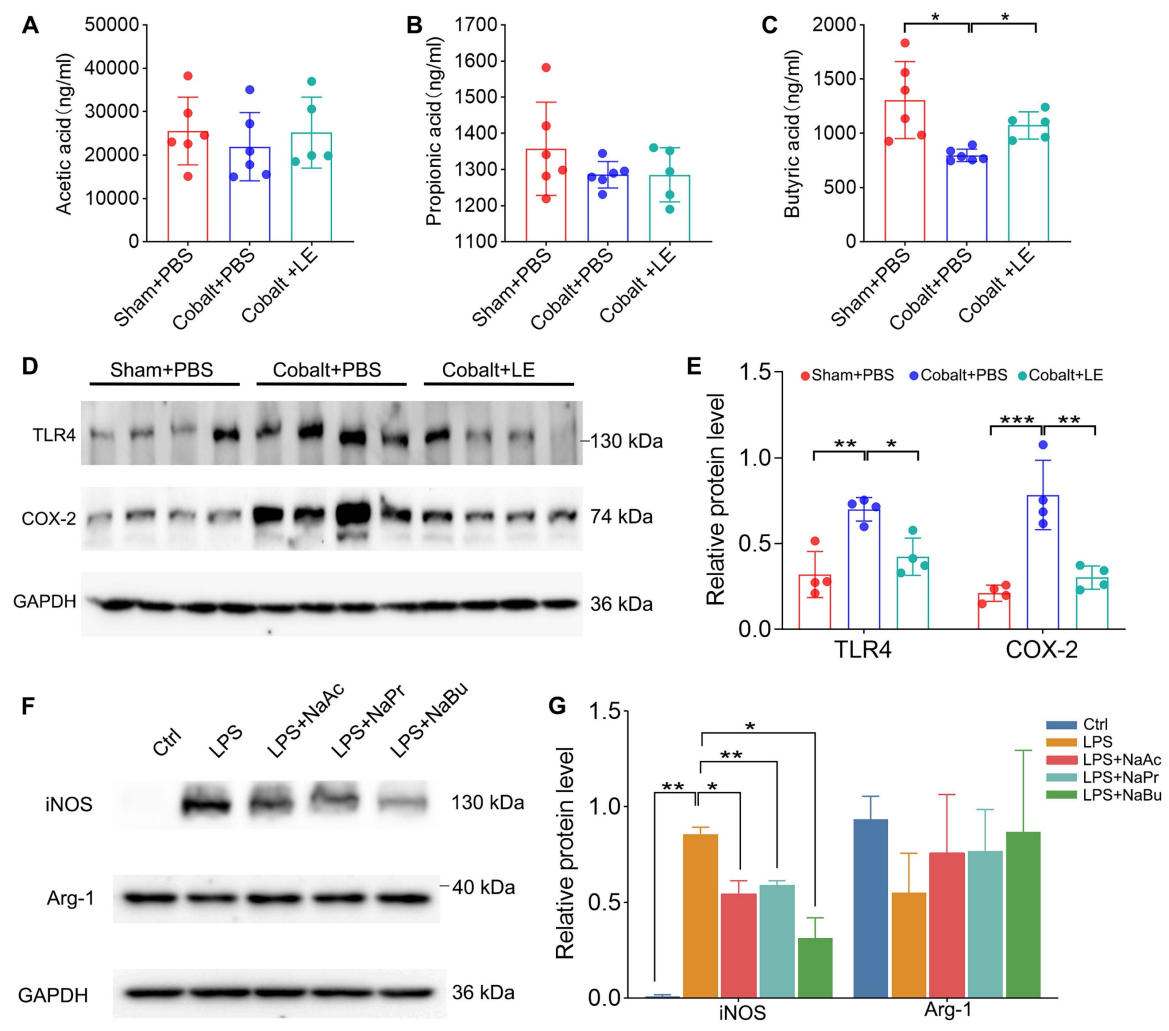
***L. eligens* elevates SCFAs *in vivo*, and SCFAs reduce iNOS in LPS-treated BV2 microglia.** (A) Acetate concentrations in each group. (B) Propionate concentrations in each group. (C) Butyrate concentrations in each group. (D) Western blot analysis of TLR4 and COX-2 expression in cortical tissues from Sham + PBS, Cobalt + PBS and Cobalt + LE groups. (E) Quantification of cortical TLR4 and COX-2 expression by grayscale densitometry using ImageJ software. (F) Representative Western blot analysis of iNOS and Arg-1. (G) Quantification of iNOS and Arg-1 expression by grayscale densitometry using ImageJ software. Data are presented as mean ± SD. One-way ANOVA followed by Bonferroni (E) and Dunnett's T3 post hoc test (C, G) was used. *p < 0.05, **p < 0.01, ***p < 0.001, vs. the Cobalt + PBS group or LPS group.

Targeted SCFAs analysis showed that, compared with the PBS-treated epilepsy group, butyrate levels were significantly increased in the *L. eligens*-treated epilepsy group (Figure 7C). Moreover, the expression of TLR4 and COX-2, which were markedly upregulated in the PBS-treated epilepsy group, was significantly reduced following *L. eligens* treatment (Figure 7D-E). To further assess the direct effects of SCFAs on microglial activation, BV2 cells were pretreated with sodium acetate (NaAc), sodium propionate (NaPr), or sodium butyrate (NaBu) for 1 h prior to 6 h of LPS stimulation. Among the SCFAs tested, butyrate exerted the strongest effect, significantly attenuating the expression of the M1 pro-inflammatory marker iNOS (Figure 7F-G).

## Discussion

In this study, we investigated the role of gut microbiota in epileptogenesis using a cobalt wire-induced rat model of chronic epilepsy. The intracortical cobalt-wire model recapitulates latent period, spontaneous recurrent seizures (focal onset with secondary generalization), neuronal loss, gliosis, neuroinflammation, and BBB alterations.

Using this model, we revealed several novel and interconnected findings that advance the current understanding of the gut-brain axis in epilepsy. Firstly, we observed a significant reduction in the abundance of *Lachnospira* during the early phase of epileptogenesis, prior to seizure onset. Not only does this temporal pattern indicate its potential as a predictive microbial biomarker, but it also supports the innovative concept that such microbial changes may be an upstream driver of epileptogenesis rather than merely a downstream consequence of seizure-induced impairment. This finding challenges the conventional notion that intestinal dysbiosis arises solely as a consequence of seizures, instead suggesting that microbial alterations may serve as a crucial predisposing factor in epileptogenesis. Secondly, we observed that oral gavage with *L. eligens* could significantly reduce the severity of seizures and prolong the latency of seizures. Furthermore, at the mechanistic level, we demonstrated that *L. eligens* could play a protective role in the occurrence of epilepsy by restoring the integrity of the intestinal barrrier and BBB, reducing circulating LPS levels, selectively inhibiting M1 microglia polarization, and down-regulating the expression of various pro-inflammatory cytokines, mediated by increased production of the SCFA butyrate, thus suppressing TLR4 signaling.

### *Lachnospira* reduction as a predictive biomarker of epileptogenesis

We observed an early decline of *Lachnospira* preceding electroclinical seizures in the animal model, accompanied by reduced butyrate and heightened pro-inflammatory signaling. This temporal pattern suggests that *Lachnospira* (or broader butyrate-producing taxa) could act as an upstream microbial signal of epileptogenesis. While causal primacy requires further testing, the alignment between microbial dynamics, SCFA availability, and neuroinflammation provides a biologically plausible framework for risk stratification.

### Alignment with clinical gut microbiota data

Consistent with the temporal signal observed in our model—i.e., an early decrease of *Lachnospira* together with butyrate depletion and microglial M1 activation—several human cohorts report a relative reduction of butyrate-producing taxa in epilepsy, most commonly at the *Lachnospiraceae* level and, in some datasets, at the *Lachnospira* genus level 37. Reports in adult focal epilepsy (including temporal lobe epilepsy) and in pediatric infantile epileptic spasms similarly note lower *Lachnospiraceae*/*Lachnospira* abundance in patients versus healthy controls, aligning with our observation that *Lachnospira* declines prior to overt seizures in the model 37-39. We acknowledge heterogeneity across studies (age, diet, geography, antiseizure medications, analytic pipelines) and therefore frame this cross-species concordance as supportive rather than definitive. As a clinical bridge, we propose prospective analyses relating *Lachnospira*/*Lachnospiraceae* and circulating/fecal SCFAs to electrographic metrics and seizure outcomes.

Previous studies in epilepsy patients and animal models also observed a reduction of *Lachnospira*, although they mostly lacked dynamic analysis, particularly before seizure onset 15, 40-42. Our results bridge this gap by showing that *Lachnospira* abundance declines significantly during the epileptogenesis stage (day 3 post-cobalt wire implantation), a period in which animals have yet to display seizure onset. This suggests that *Lachnospira* reduction could be among the earliest microbial shifts that make the host significantly more susceptible to epilepsy.

The implications of this time-windowed signature are substantial. It positions *Lachnospira* as a potential microbial biomarker for identifying individuals at risk of epilepsy before clinical onset, thereby opening opportunities for early intervention. This aligns with Hill's criteria for causality, including temporality and biological plausibility, and reflects emerging views that gut microbial shifts can not only reflect but also influence CNS disease trajectories 43.

### *L. eligens* supplementation mitigates seizure severity and delays onset

Functionally, oral gavage supplementation with *L. eligens* for 15 days led to stable gut colonization and significantly reduced the severity of generalized seizures (Racine stages 4-5) while prolonging seizure latency. These findings indicate a tangible neuroprotective effect, particularly in mitigating the transition from partial to generalized seizures. While prior studies have demonstrated the anti-seizure effects of other probiotics such as *Lactobacillus* and *Bifidobacterium* 44-46, these effects have largely been attributed to general anti-inflammatory or gut barrier-enhancing properties. In contrast, our results uniquely identify *L. eligens* as a modulator of epileptogenesis.

This early-phase modulation of seizure expression further substantiates *Lachnospira's* involvement in epileptogenesis, rather than merely seizure propagation or chronic sequelae. The suppression of high-grade seizures in the absence of changes to low-grade seizure frequency suggests that *L. eligens* may dampen epileptic network synchronization and excitability thresholds to prevent seizure generalization. This positions it as a novel candidate for disease-modifying intervention, rather than symptomatic control alone.

### Multi-barrier protection: *L. eligens* reinforces gut and brain barriers

Mechanistically, our findings support that *L. eligens* reinforces both intestinal barrier and BBB integrity. Enhanced intestinal barrier function was directly confirmed by the FD-4 assay. Occludin, a tight junction protein critical to epithelial and endothelial barrier function 47, was significantly upregulated in both colonic and cortical tissues following* L. eligens* treatment. Notably, the enhancement of the intestinal barrier coincided with a marked reduction in serum LBP levels, suggesting that improved gut barrier function effectively restricted endotoxin translocation and mitigated systemic inflammatory responses. Additionally, the restoration of BBB integrity was associated with decreased expression of proinflammatory cytokines and microglial activation in the cortex, implying that *L. eligens* indirectly protects the brain.

Previous studies have demonstrated that gut microbiota influence BBB permeability via microbial metabolites, particularly SCFAs like butyrate and acetate 18, 48, 49. *L. eligens* is a specific SCFA-producing taxon, and consistent with this, our serum analysis showed that supplementation with *L. eligens* significantly increased butyrate levels, providing a direct link between microbial metabolites and barrier stabilization. These findings are consistent with previous reports showing that germ-free animals exhibit compromised BBB integrity, which can be restored upon microbial colonization, highlighting the critical role of gut microbiota and their metabolites, such as SCFAs, in maintaining barrier function 48, 49.

In the context of epilepsy, barrier dysfunction has been shown to exacerbate neuroinflammation and facilitate seizure generation 50, 51. The dual-barrier stabilizing effect of *L. eligens* may play a central role in its antiepileptic efficacy. This suggests that *Lachnospira* operates not solely through microbial competition or niche occupation but engages in metabolite-mediated immunological regulation.

### Neuroimmune modulation through SCFA-mediated suppression of microglial M1 polarization

A key downstream effect of L. eligens supplementation was the suppression of cortical microglial activation and skewing away from pro-inflammatory M1 polarization. Immunofluorescence and quantitative PCR data showed reduced expression of CD68 and iNOS, as well as significant downregulation of pro-inflammatory cytokines including IL-1β, IL-6, and TNF-α. Previous studies have shown that these cytokines are well-characterized inducers of neuronal hyperexcitability and have been implicated in seizure exacerbation through modulation of glutamatergic and GABAergic transmission 52-54.

Our findings align with and extend recent work implicating M1-polarized microglia in epileptogenesis through TLR-mediated NF-κB and STAT3 signaling 55-57. Activation of the TLR-NF-κB-STAT3 axis initiates a cascade of transcriptional responses that amplify neuroinflammation, promote microglial proliferation, and sustain the expression of pro-convulsant cytokines. Importantly, our results showed that supplementation with *L. eligens* significantly reduced cortical TLR4 and COX-2 expression, along with downregulation of IL-1β, IL-6 and TNF-α, both direct transcriptional targets of NF-κB, indicating upstream inhibition of this pro-inflammatory signaling pathway. Furthermore, reduced iNOS expression, a STAT3-responsive gene associated with oxidative stress and excitotoxicity, supports the interpretation that both branches of this pro-inflammatory axis are functionally suppressed.

Although L. eligens did not markedly upregulate M2 marker expression, its selective inhibition of the M1 axis suggests a regulatory effect on microglial phenotype that restores immune homeostasis without inducing generalized immunosuppression. This nuanced immunomodulation may be particularly beneficial in chronic neuroinflammatory conditions such as epilepsy 58.

Mechanistically, the neuroprotective and immunomodulatory effects of *L. eligens* appear to be largely mediated by its metabolic production of SCFAs, especially butyrate. Our *in vitro* experiments demonstrated that SCFAs, especially butyrate, directly inhibit M1 microglial, indicating that SCFA-mediated immunomodulation is a key mechanism underlying the antiepileptic effects of *L. eligens*.

Taken together, these observations place *L. eligens* at the nexus of gut-derived metabolite signaling, systemic inflammation, and central neuroimmune activation—an intersection increasingly recognized as critical in the pathogenesis of neuroinflammatory diseases, including epilepsy. Supporting this notion, supplementation with *Lactobacillus helveticus* R0052 has been shown to increase SCFAs, particularly butyrate, and to elevate seizure thresholds in mice 59.

### Therapeutic implications and future perspectives

The multifaceted effects of *L. eligens*—including microbial restoration, barrier reinforcement, and immunomodulation—highlight its potential as a prototype for microbiota-based therapeutic strategies in epilepsy and other neuroimmune disorders. Unlike conventional antiepileptic drugs that primarily target neuronal ion channels or neurotransmitter systems, microbiota-based therapies offer the promise of modulating upstream triggers of disease, including inflammation and barrier dysfunction. These findings reinforce the paradigm that microbiota are not passive reflectors of disease state but active participants in CNS pathophysiology.

Current microbiota strategies such as the ketogenic diet (KD) have shown efficacy in reducing seizures and modulating the gut-brain axis. However, its restrictive nature and potential metabolic side effects often limit long-term adherence 60. In this context, *L. eligens* offers distinct advantages. Importantly, just like other probiotics, it has been shown in our animal models to generally exhibit higher safety and tolerability, which may enhance patient adherence when used alone or alongside conventional medications. Notably, preclinical studies have shown that probiotics can complement conventional antiepileptic drugs; for example, probiotics combined with pregabalin or brivaracetam mitigated seizures, neurobehavioral deficits, and neurodegeneration in PTZ-kindled mice 61, 62. These findings suggest that *L. eligens* could be developed into formulations such as capsules, beverages, or liquid suspensions to maintain bacterial viability and optimize delivery. It may also function both as a stand-alone therapy and as an adjuvant, leveraging its safety profile and adherence benefits.

Further exploration of *Lachnospira* metabolites, such as SCFAs and other immunomodulatory molecules, may help elucidate precise molecular mechanisms. To this end, studies could include: (i) pharmacological interference of FFAR2/FFAR3 or TLR4 signaling (antagonist/inhibitor or siRNA) to test reversibility; (ii) conditioned medium versus heat-killed bacteria controls to distinguish metabolite- versus cell-associated effects; and (iii) readouts of NF-κB p65 nuclear translocation and HDAC activity/histone acetylation, consistent with known butyrate actions. Additionally, future studies employing gnotobiotic models or microbiota transplantation will be instrumental for establishing causality and for translation to human epilepsy.

### Limitations and future directions

We acknowledge that the cobalt-wire model initiates seizures focally and may under-represent multifocal/network-wide inflammation. To strengthen generalizability, we will conduct cross-model validation in chemoconvulsant paradigms (pilocarpine and kainic acid) that exhibit broader inflammatory tone and multifocal network involvement. These studies will test whether *L. eligens*/SCFA-centered interventions reproduce the observed effects on seizure burden, microglial activation (iNOS), gut permeability (FD-4), and systemic inflammatory readouts (e.g., LBP/cytokines), and will compare outcomes across models to refine translational relevance. We also note that qPCR alone cannot distinguish live versus dead bacteria or persistence after cessation; as next steps, PMA-qPCR, culture, or metagenomic confirmation and post-cessation follow-up will be conducted.

## Conclusions

Taken together, our study provides compelling evidence that gut microbiota plays a crucial role in epileptogenesis and epilepsy development. Supplementation with *L. eligens* effectively alleviates epilepsy symptoms by modulating gut microbiota composition, restoring BBB integrity, and suppressing neuroinflammation. These findings highlight the therapeutic potential of gut microbiota modulation in epilepsy management and open new avenues for microbiota-based interventions in neurological disorders.

## Supplementary Material

Supplementary table.

## Abbreviations

EEG: electroencephalogram
BBB: blood-brain barrier
*Lachnospira* eligens: L. eligens
LPS: lipopolysaccharide
ELISA: enzyme-linked immunosorbent assay
IL-1β: interleukin-1β
IL-6: interleukin-6
TNF-α: tumor necrosis factor-α
CNS: central nervous system
TLR: Toll-like receptor
SCFAs: short-chain fatty acids
SPF: Specific Pathogen**-**Free
DAB: diaminobenzidine
