# Supplementary material for: Lachnospira eligens attenuates epileptogenesis via gut-brain axis regulation of blood-brain barrier integrity and neuroinflammation
Source: Theranostics. 2026 Jan 1;16(2):1045–62. doi: 10.7150/thno.116959 (PMC12675144; doi:10.7150/thno.116959)
Supplement: Supplementary file 1 — Supplementary table. [file thnov16p1045s1.pdf]

Supporting Information

Lachnospira eligens attenuates epileptogenesis via gut-brain axis regulation of blood-brain barrier integrity and neuroinflammation

Huifeng Li[1, 2], Ruili Niu[1, 2], Wenzhen He[3], Huanling Lai[1, 2], Shangnan Zou[4], Qihang Zou[5], Yue Gui[1, 2], Tengyue Zhang[1, 2], Guoyun Feng[2, 5], Yue Xing[1, 2], Dehai Gou[2], Xiaofeng Yang[1, 2, 3] *

1. Department of Neurology, The First Affiliated Hospital, Guangzhou Medical University, Guangzhou 510120, China.

2. Department of Basic Research, Guangzhou National Laboratory, Guangzhou 510005, China.

3. Department of Neurology, The First Affiliated Hospital of Shantou University Medical College, Shantou 515041, China.

4. Department of Neurology, Sun Yat-sen Memorial Hospital, Sun Yat-sen University, Guangzhou 510120, China.

5. Department of Neurology, The Seventh Affiliated Hospital, Sun Yat-sen University, Shenzhen 518107, China.

* Corresponding author: Xiaofeng Yang, MD.

Address: Guangzhou National Laboratory, No. 9 XingDaoHuanBei Road, Guangzhou International Bio Island, Guangzhou 510005, Guangdong Province, China.

Email: yang_xiaofeng@gzlab.ac.cn.

Table S1 Primer sequences used for qPCR

Gene	Primer sequences (5'-3')
L. eligens	F: CTGACTTGCATGGCCACCTA
	R: GGCTAAATTCGTGCCAGCAG
Universal 16S	F: AGAGTTTGATCCTGGCTC
	R: TGCTGCCTCCCGTAGGAGT
IL-1β	F: AATCTCACAGCAGCATCTCGACAAG
	R: TCCACGGGCAAGACATAGGTAGC
IL-6	F: ACTTCCAGCCAGTTGCCTTCTTG
	R: TGGTCTGTTGTGGGTGGTATCCTC
TNF-α	F: CTCTTCTCATTCCTGCTCGT
	R: CATTTGGGAACTTCTCCTCCT
IL-10	F: TGGGAGAGAAGCTGAAGACC
	R: TGGCCTTGTAGACACCTTTG
GAPDH	F: GACATGCCGCCTGGAGAAAC
	R: AGCCCAGGA TGCCCTTTAGT
